# *Chlamydia abortus* Isolation and Identification in Aborted Ovine Fetus in Mari El Republic of Russia

**DOI:** 10.3390/pathogens11121408

**Published:** 2022-11-24

**Authors:** Irina Matveeva, Nikolai Nikitin, Ekaterina Evtushenko, Karim Azimov, Alexey Zaberezhny, Olesya Bogomolova, Maria Kruglova, Vladimir Yeremets, Evgeniya Markova, Natalia Yeremets

**Affiliations:** 1 All-Russian Scientific Research and Technological Institute of Biological Industry, Biocombinat, 141142 Moscow, Russia; 2 Department of Virology, Lomonosov Moscow State University, 119234 Moscow, Russia

**Keywords:** chlamydia, ovine enzootic abortion, outer membrane protein A (OmpA), isolates

## Abstract

Reproductive disorders, presumably caused by *Chlamydia abortus*, are common among the ovine population of the Mari El Republic, Russia. *C. abortus* infection was determined by serologic testing or isolation and detection of the organism by PCR and direct immunofluorescence in tissue samples. Rams, ewes, and lambs (10 individuals each) were randomly chosen for serological testing by the complement fixation test and 7 of 30 (23%) animals tested were positive. Tissue samples were collected from ewes and aborted fetuses for isolation by inoculating chicken embryo yolk sacs (*n* = 41). The same samples were analyzed by PCR using commercial and in-house PCR kits and by direct immunofluorescence. *C. abortus* was detected in 58.5% of samples using PCR and in 60.9% of the samples by direct immunofluorescence. Five *Chlamydia* isolates were cultured in egg yolk sacs and adapted for growth in cell cultures. Phylogenetic analysis showed no substantial difference between Russian isolates and those from other parts of the world. The results of the study further demonstrate the usefulness of PCR for detection of *C. abortus* as a faster, simpler, and more reliable approach in comparison to culturing the organism and underscoring the necessity of screening for chlamydiosis as a cause of ovine abortion.

## 1. Introduction

*Chlamydia abortus* (family: *Chlamydiaceae*) is a non-motile, coccoid, pleomorphic, Gram-negative obligate, intracellular bacterium that causes disease in both humans and animals [1,2]. An economically important disease caused by *Chlamydia abortus* in numerous sheep-breeding areas worldwide is the enzootic abortion of ewes (EAE) [3,4,5]. 

This disease can present in a variety of clinical manifestations: endometritis; infertility; premature delivery of weak lambs; third-term abortion; perinatal death; orchitis; and seminal vesiculitis. Pneumonia, enteritis, encephalomyelitis, conjunctivitis, and polyarthritis are other symptoms of EAE [6]. Infection tends to occur oral–nasally with infected aborted fetal tissues, placentas, and uterine discharge the main sources of infection. *C. abortus* elementary bodies remain infective in the ambient environment for several days in typical spring weather, and for several months if the temperature is low or close to 0 °C. Based on available data, chlamydial infection is the leading etiological abortion factor in small ruminants, which is responsible for 23–39% of cases [7], however, clinical identification of EAE is often hindered by concurrent infections with other abortive agents, such as *Campylobacter* sp., *Toxoplasma* sp., *Listeria* sp., *Brucella* sp., *Salmonella* sp., and the border disease agent [8,9,10].

The worst damage caused by chlamydia to cattle breeding in Russia is attributed to abortions, the delivery of stillborn or weak offspring, and, to a lesser extent, cases of bronchopneumonia, arthritis, keratoconjunctivitis, and encephalitis. However, very few studies of EAE have been carried out in Russia [11,12], however, some regions have documented several cases of ovine chlamydiosis. Therefore, we focused on the Mari El Republic which had a history of urogenital tract pathologies, abortions, stillbirths, or delivery of weak lambs. The purpose of the study was therefore to isolate, identify, and perform a molecular genetic testing of *Chlamydia abortus* isolates from ewes in the Mari El Republic to find their phylogenetic position in the *Chlamydiaceae* family compared to isolates with other chlamydia strains deposited in GenBank, using the outer membrane protein A (*OmpA*) gene as a reference.

## 2. Materials and Methods

### 2.1. Study Area and Sample Collection

Pregnant sheep (*n* = 10), stud rams (*n* = 10) and lambs two to three months old (*n* = 10) were selected randomly for serologic testing for *C. abortus* (using a CFT, VNITIBP, Russia)*, Brucella* spp. (using ID Screen Brucellosis Serum Indirect Multi-species; ID-VET, France), *Toxoplasma gondii* (using ID Screen Toxoplasmosis Indirect Multi-species; ID-VET, France), *Coxiella burnetii* (using ID Screen Q Fever Indirect Multi-species; ID-VET, France), and *Maedi-Visna* virus (using ID Screen MVV/CAEV Indirect; ID-VET, France) before beginning to extract *C. abortus* isolates. In practical terms, the samples were collected as follows: paired sera were sampled from 10 pregnant sheep on day 1 or 2 after stillbirth and three weeks later.

Moreover, we collected 12 specimens of aborted fetuses from 12 non-vaccinated sheep in five flocks located in different districts of the Mari El Republic. Each flock had more than 100 ewes which had not been vaccinated in the past 10 years. Figure S1 (Supplementary Materials) displays the locations of the five sheep flocks selected for the study on the map of the Mari El Republic. 

In order to isolate *chlamydia*, the following tissue samples were used (*n* = 41): cotyledons and placental membranes of an infected animal; vaginal swabs; liver, spleen and lungs of a fetus collected with the help of disposable razors and scissors. Samples for planned tests were put in plastic containers, labeled, and stored in a refrigerator box. 

Vaginal swabs collected for nucleic acid extraction were transferred to 200 μL lysis buffer (6 M guanidium isothiocyanate, 10 mM urea, 20% (*v*/*v*) Triton X-100 and 10 mM Tris HCl (pH 4.4); Roche Diagnostics, Mannheim, Germany) and stored at 4 °C. Afterbirths and organ samples from aborted fetuses were stored at −80 °C until further use [13].

The study on animals was approved by the Commission on Bioethics of All-Russian Scientific Research and Technological Institute of Biological Industry (protocol No 17 dated 12.01.2021).

### 2.2. Cell Cultures and Chicken Embryos

The study involved 6–8 day old chicken embryos and a McCoy cell line (ATCC, catalog number CRL-1696). The mouse-derived McCoy fibroblasts cell line and DMEM with high glucose and L-Glutamine were purchased from the American Type Culture Collection (ATCC, Manassas, VA, USA).

### 2.3. Collection and Preparation of Specimens for Extracting C. abortus Isolates

Samples of ovine placenta tissues (*n =* 12); vaginal swabs (*n =* 5) and aborted fetus organs (*n =* 12): lungs (*n =* 12), liver (*n =* 5), and spleen (*n =* 7), were collected (*n =* 41) and processed in the following way. Tissue homogenization was performed by pulverization using a sterile pestle and mortar. Homogenized tissues were suspended in the DMEM growth medium containing L-glutamic acid (4.9 mM), fetal bovine serum (10%), streptomycin (100 μg/mL), gentamicin (50 μg/mL), and nystatin (50 μg/mL) to obtain a 10% suspension. The suspension was centrifuged at 2000× *g* for 10–15 min. After that, the supernatants were collected and aliquoted in small volumes. One part of the supernatant was used directly for *C. abortus* isolation on chicken embryos and on the McCoy cell culture, and the other part was stored at −70 °C. 

Vaginal swabs collected for subsequent cell culture cultivation were transferred to 1 mL of sucrose phosphate glutamate buffer (SPG, 218 mM sucrose, 38 mM KH_2_PO_4_, 7 mM K_2_HPO_4_, 5 mM L-glutamic acid) supplemented with 0.1% (*v*/*v*) bovine serum albumin V, and stored at −80 °C [13].

### 2.4. Chlamydia Abortus Isolation

For the purpose of *C. abortus* isolation, yolk-sac membranes of developing chicken embryos were inoculated with tissue suspension (0.2 mL) of aborted animal fetuses and placentas and incubated at 37 °C. Infected embryos would die 4 to 13 days after inoculation.

As soon as the embryos started dying after inoculation, yolk-sac membranes were immediately removed from dead or dying embryos and homogenized by shaking in flasks containing glass balls. Each suspension was titrated by enumerating inclusion-forming units (IFU) in McCoy cells under the microscope (Olympus CKX53, Tokyo, Japan). The suspensions containing high concentrations of chlamydia inclusions were put together and clarified by low-speed centrifugation in order to remove the cell and yolk remnants. The ready yolk-sac emulsion was used as the inoculum for further passages.

Inoculum titration was performed both on chicken embryos, expressing infection activity in ELD_50_/mL (50% embryo lethal dose), and by enumerating the inclusion-forming units (IFU) in McCoy cells, and then the standardized aliquots were frozen at −80 °C until use. 

Further adaptation of *Chlamydia* isolates was performed by direct successive passaging on the McCoy cell culture and chicken embryo yolk sacs. 

McCoy cells were suspended in the DMEM growth medium with 10% fetal bovine serum and antibiotics (as described above) at a concentration of 2 × 10^5^ cells/mL. Aliquots of 2 mL of cell suspension were spread across flat-bottomed matrasses containing cover glasses. After incubation for 24 h at 37 °C, a confluent culture monolayer was obtained. The growth medium for cells was removed and replaced with the test material in order to inoculate the cells. After inoculation with the test material, the flasks were incubated for 2 h, then the inoculating liquid was drained and replaced with the DMEM growth medium containing antibiotics, but no serum. Then, the cells were incubated for another 18–24 h, after which the medium was renewed again, and the incubation continued for seven days. Three to five days after inoculation, one cover glass was fixed in methanol and put to a qualitative direct immunofluorescence assay in order to confirm the presence of chlamydia (inclusions) in the cell culture. The cultured cells that turned out negative seven days after inoculation were passaged again using the same method as described above (up to three blind passages).

### 2.5. Detection of the Chlamydia via Direct Immunofluorescence Assay

A McCoy cell culture monolayer or imprint smears of chicken embryo yolk sac suspension were stained with a reagent provided as part of the Fluorescent Globulin Set for Diagnosing Chlamydia Infection in Animals (VNITIBP, Russia). *Chlamydiaceae-*specific sheep fluorescent antibodies recognizing surface lipopolysaccharides of all known *Chlamydia* species are used in this set. A specimen was considered positive when inclusions of typical chlamydial morphology manifested themselves as bright apple-green spots after two passages. 

The immunofluorescence background was eliminated using the addition of 5% BSA dissolved in PBS (pH 7.2). In short, monolayers of infected cells or smear stains were fixated with cold methanol for 10 min, then washed and treated with 5% BSA. Samples were incubated for 1 h at 37 °C in humid chamber. Fluorescent globulin was diluted 1/200 in PBST (PBS + 0.2% Tween 20) with the addition of 1% BSA and then incubated with the samples for 1 h at 37 °C. After tri-fold washing, the infected cells were observed with a fluorescent microscope CKX53 (Olympus, Japan) at magnification 40 × 0.3. Uninfected cells or uninfected yolk sacs’ smear stains were used as a negative control.

### 2.6. Detection of the Chlamydia by Complement Fixation Test

Immunological detection of specific anti-chlamydia antibodies in serum over time (paired sera method) was carried out using the set of components for chlamydia infection diagnosing through a complement fixation test (CFT) or prolonged complement fixation test (PCFT) (VNITIBP, Russia).

The CFT detects antibodies to *Chlamydia* genus-specific antigens and can be used to detect all chlamydial infections. This is because the chlamydial antigen contains LPS as an immunodominant component, which is common to all *Chlamydiaceae* species. CFT testing was carried out in 96-well ‘U’ bottom microtiter plates (Thermo Fisher Scientific, Waltham, MA, USA). Samples are tested at twofold dilutions from 1 to 5. A dilution of sera in Kolmer diluent (150 mM NaCl; 0.2 mM CaCl_2_; 0.63 mM MgCl_2_; 0.2 mM sodium azide) were tested following inactivation by heating at 60 °C for 45 min in an incubator. The inactivated and diluted sera were then mixed with *Chlamydia abortus* antigen and guinea pig complement diluted to 1:50 and 1:60, respectively, in Kolmer diluent and incubated at 37 °C for 1 h. Twenty-five microliters of hemolysin-sensitized 3% sheep red blood cells (RBCs) were then added to the serum/antigen/complement mixture and incubated at 37 °C for 30 min on a plate shaker (Orbital Shaker-incubator ES-20; Biosan, Latvia) at 1000–1200 rpm. Following incubation, plates were centrifuged at 524× *g* and the results read on a light box. Inhibition of hemolysis demonstrated the presence of specific antibody while complete hemolysis indicated the absence of detectable levels of specific complement-fixing antibodies. The reciprocal of the highest dilution of serum showing ≤50% (2+) hemolysis indicated the antibody titer of the serum. Reactions of 2+ or greater at a dilution ≥ 1:10 were considered positive.

### 2.7. Chlamydia abortus DNA Detection

Collected samples were used to detect the DNA of *C. abortus* through a real-time PCR test using the Kylt^®^ *Chlamydia abortus* Detection Kit, AniCon, Germany, in line with the manufacturer’s instructions. 

Vaginal swabs collected for nucleic acid extraction were transferred to 200 μL lysis buffer (6 M guanidium isothiocyanate, 10 mM urea, 20% (*v*/*v*) Triton X-100, and 10 mM Tris HCl (pH 4.4); Roche Diagnostics, Mannheim, Germany) and stored at 4 °C. Afterbirths and organ samples from aborted fetuses were stored at −80 °C until further use [13]. 

The QIAamp DNA Mini Kit (Qiagen, Germany) was used for DNA extraction from swabs (or suspension) of chicken embryo yolk sacs, aborted fetus organs or McCoy cell culture monolayer in line with the manufacturer’s instructions. DNA extracts were stored at −20 °C before analysis. Extraction of *Chlamydia* isolates by inoculation of chicken embryos and the cell culture was reconfirmed by in-house PCR based on the partial amplification of the *OmpA* gene sequence region (middle 80% of the gene) that is 210 base pairs (bp) long using two primers by LLC New Molecular Technologies, Russia: omp-F: *GATGGCACTATGTGGGAAGGT*, and omp-R: TTGGGTTCCATGTGGTCAAGT. SYBR Green I (Thermo Fisher Scientific, USA) was used as a dye.

In-house PCR test was conducted as follows: The PCR mixture consisted of 1 U Taq-DNA-polymerase, 1.5 mM magnesium chloride, 200 μM dinucleotide triphosphates, and a normal PCR buffer. Amplification was performed using the CFX96 Touch Real-Time PCR Detection System (BioRad) in line with the following protocol: 94 °C for 4 min, followed by 40 cycles of heating to 94 °C for 1 min and cooling to 59 °C for 1 min, with the final fill-in at 72 °C for 2 min. The DNA of a bacterium from a commercial vaccine (Chlamydiovac Armavir Biofactory, Russia) was used as a positive control. Reagents of the PCR mixture without the matrix DNA were used as a negative control. 

### 2.8. OmpA Gene Sequencing and Phylogenetic Analysis

The *OmpA* gene tested in an in-house PCR test was then used for locus phylogenetic analysis of *C. abortus* isolates. Omp-F and omp-R primers were used for the amplification of the 1058 bp-long region in order to obtain a larger product of the *OmpA* gene for sequencing, as executed before [14]. 

DNA sequences were determined by the direct sequencing of PCR products using the ABI Prism 3100 Genetic Analyzer (Applied Biosystems, Foster City, CA, USA) and the BigDye Terminator v3.1 Cycle Sequencing Kit (Applied Biosystems, Foster City, CA, USA) in line with the manufacturers’ instructions.

Phylogenetic placement of chlamydia strains in the *Chlamydiaceae* family and phylogenetic tree construction on the basis of *OmpA* gene sequencing results were performed in the MEGA 11 software using the Neighbor-Joining method and p-distance model alongside with the Bootstrap Test of Phylogeny (1000 replicates) and the Maximum Parsimony method [15]. The homology of sequences of tested strains and various chlamydia types available in NCBI GenBank was measured using the BLAST software. MEGA 11 was also used to evaluate evolutionary divergence between the sequences and the standard mean square error(s). Alignment was performed with the help of the ClustalW algorithm.

## 3. Results

During the 2021 breeding season, from March to May, abortions in sheep flocks were reported in several districts of the Mari El Republic. The highest number of cases was detected at farms in the Medvedevo district. According to information obtained from the farms, their sheep had also had abortions during prior breeding seasons. The sheep flock management system in all the districts of the Republic is classic: the flocks had different owners; the sheep were fed with grain, hay and silage and left to graze on a pasture. Different animal species would often graze together on the same pasture. Sheep farmers in those districts bred local breeds or the Romanovskaya breed. As a rule, an abortion at their farms would occur in the last 2–3 weeks of pregnancy and be notable for stillbirth and placentitis. Moreover, the delivery of fully developed stillborn lambs or weak living lambs that were viable for up to 48 h was occurring. Ewes would give birth to healthy lambs as well, and the simultaneous delivery of a stillborn and a living weak/healthy lamb was frequently observed.

In order to establish the ethological role of chlamydia in the sheep pathology, we performed a set of laboratory tests that included serum testing for specific antibodies, chlamydia antigen detection in the pathological material using the immunofluorescence assay, agent isolation on developing chicken embryos, and a cell culture, as well as real-time PCR. The grounds for recognizing *Chlamydia* isolates and the presence of an infection caused by *C. abortus* were: recent abortions in the medical history of ewes (often at late pregnancy stages) and detection of necrotic inflammation of the placenta and high concentrations of agent DNA via real-time PCR testing of clinical specimens. Typically, the infection is represented with a characteristic thickening and reddening of the intercotyledonary membranes due to edema, with inflamed cotyledons, and often a creamy exudate on the surface of the membranes. This differs from, for example, toxoplasmosis, another common cause of abortion, where placental membranes are largely unaffected, while cotyledons demonstrate white foci of necrosis [9].

Prioritization of sample screening on different infectious agents, causing abortions in sheep, depended on availability of certain diagnostic kits. In the end, serological screening for *Coxiella burnetii*, *Brucella* spp., Maedi-Visna virus, *Chlamydia abortus*, and *Toxoplasma gondii* was performed in order to evaluate the presence of these pathogens. No such abortive pathogens as *Coxiella burnetii*, *Brucella* spp., Maedi-Visna virus, and *Toxoplasma gondii* were detected in the sheep selected for the experiment. It was established that the random serological testing of paired sera of ewes after an abortion or stillbirth showed an increase in the specific chlamydia antibody titer. In particular, complement fixation tests with a chlamydial antigen were positive for 7 (23%) out of 30 examined animals of various age-sex groups. The results of these studies are presented in Table 1. Lambers turned out the most infected group, followed by lambs and stud rams. 

The results of serologic testing were confirmed by chlamydia isolation from the pathologic material of aborted fetuses and sheep placenta in a bioassay on chicken embryos and a cell culture. An immunofluorescence assay detected chlamydia in the pathological material in 60.9% of cases, and a PCR test in 58.5% of cases (Table 2). Of 25 samples confirmed positive in the immunofluorescence assay, 24 were also confirmed positive via PCR test. All samples isolated in chicken embryos were positive in two other methods except for one sample, which was positive in immunofluorescence analysis but negative in real-time PCR.

After several series of passages, 5 isolates with high reproduction capability out of 41 samples of aborted fetal organs, vaginal swabs, and ovine placentas were obtained using inoculation of the chicken embryo yolk sac. 

In order to detect chlamydial antigens using the direct immunofluorescence assay, ready imprint smears were stained with FITC-labeled chlamydial antibodies, exposed and studied under a luminescent microscope. Circular structures on the positive sample staining represent chlamydial cells.

All the *Chlamydia* isolates propagated themselves in the epithelial cells of chicken embryo yolk-sacs, causing their death on day 5–10 after inoculation, with infection titers reaching 10^6.5^ ELD_50_/0.3 mL. The infected yolk sacs were thin-walled, and their blood vessels strongly occluded, which delayed embryo growth. 

Thus, clinical epizootic, serologic, and microbiologic tests produced the results making it possible to assume that the etiological agent of a disease that manifests itself through abortions and stillbirths during the mass lambing (breeding) period in studied animals is chlamydia.

For sequencing with the purpose of further molecular genetic studies of *Chlamydia* isolates from sheep in Russia and their phylogenetic placement in the *Chlamydiaceae* family, we chose the target genome fragment, the *OmpA* gene.

We performed total chlamydia DNA isolation from samples and prepared DNA libraries for sequencing. Five *Chlamydia* isolates, Chlamydia Mari El 21-1, Chlamydia Mari El 21-2, Chlamydia Mari El 21-3, Chlamydia Mari El 21-4 (named after their area of origin (Table 3)), and Chlamydia VNITIBP-21 (isolate with the most reproductive capability) were chosen for further analysis. After a series of passages on the McCoy cell culture, the sequences of the outer membrane protein A (OmpA) gene of those isolates were deposited with GenBank in line with an established procedure.

**Figure 1:**
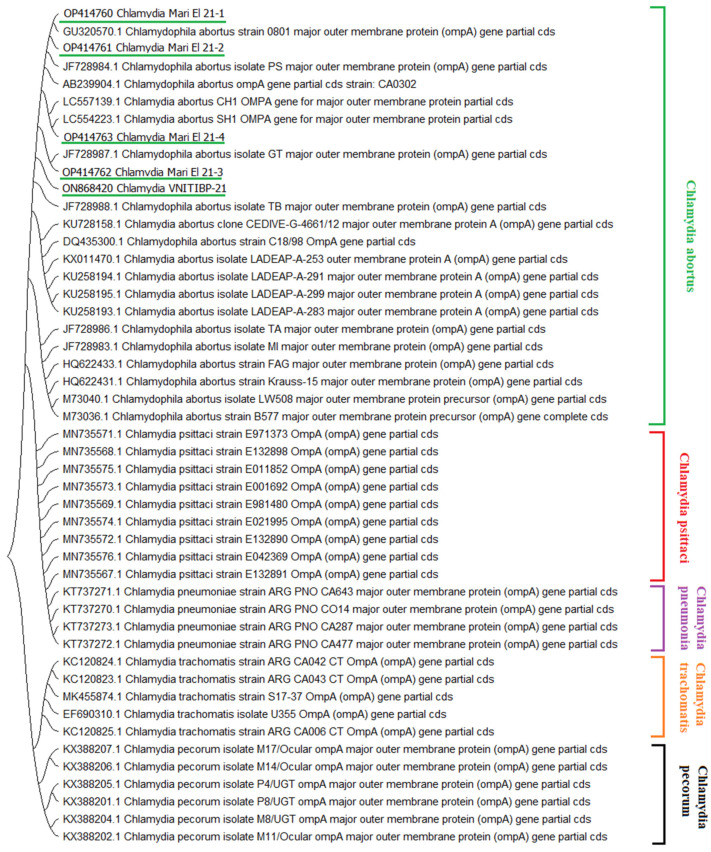
Dendrogram obtained via a phylogenetic analysis of *OmpA* gene sequences of representatives of the genus *Chlamydia*. The sequences were obtained from the GenBank and MLST (http://pubmlst.org/chlamydiales/, accessed on 15 August 2022) websites. Multiple alignments were made with the help of the Clustal W algorithm [16], and phylogenetic analysis was performed with the MEGA 11 software using the Neighbor-Joining method. The isolates obtained in this study are underlined in green.

We confirmed that the five isolates from ovine fetuses belonged to *C. abortus* of the *Chlamydiaceae* family, and placed them among the isolates whose nucleotide sequences are available in the GenBank electronic database. The phylogenetic analysis showed that all local strains were grouped together (Figure 1). Sequencing of the *OmpA* gene (outer membrane protein of *C. abortus*) of the Chlamydia VNITIBP-21 strain made it possible to estimate the homology of *OmpA* nucleotide sequences at above 99.0 to other known chlamydia strains presented in Table 4.

Topology of the phylogenetic tree based on the *OmpA* gene showed that all the *Chlamydia* isolates belong to the type *C. abortus* and have the same ancestor as ovine isolates with the accession numbers of KU728158 and JF728988. The closest analogues of Russian strain Chlamydia VNITIBP-21 are: GU320570.1 (West China); JF728984.1 (Greece); AB239904.1 (Japan); LC557139.1 (Egypt); LC554223.1 (Egypt); JF728987.1 (Greece); JF728988.1 (Turkey); KU728158.1 (Argentina); DQ435300.1 (The Netherlands); and KX011470.1 (Argentina). Our study added to the knowledge on *C. abortus*, showing that it is a highly monomorphic group with a similar content of nucleotide sequences in the *OmpA* gene.

The Chlamydia VNITIBP-21 strain turned out stable in terms of genetic characteristics of OmpA throughout 20 passages on the McCoy cell culture. To study genetic stability of Chlamydia VNITIBP-21 isolate, a series of passages with 5–7 day intervals during the course of 6 months was performed. To achieve that goal, the samples of chlamydia isolate of the 20th passage, grown on McCoy cell culture, were obtained, as well as the samples of chicken embryo yolk sac after the second passage of the isolate. The sequence of *ompA* gene of the 2nd (yolk sac) and 20th (McCoy cell culture) passage were 100% identical (873 b.p. were aligned; Figure S2). The data collected confirm the genetic stability of the *ompA* gene of Chlamydia VNITIBP-21 isolate. These data could provide valuable information for the further use of the isolate to produce vaccines and diagnostics.

## 4. Discussion

*Chlamydia abortus* is a livestock pathogen with a major economic impact as it causes ovine enzootic abortion and may also cause zoonotic infections in humans. Even though the pathogen is endemic among small ruminants, it may also infect bovine cattle, swine, deer, horses, and other hosts [17]. However, regardless of numerous reports on chlamydia infection isolation in small ruminants worldwide [18,19,20] microbiological and genetic studies of animal chlamydia infection have not been conducted in Russia.

Here, we used the complement fixation test (CFT) that the World Organization for Animal Health (WOAH) referred to as the most frequent method of animal chlamydia infection serodiagnosis. However, it is a labor-intensive method, and since *C. abortus* has the same antigens as *C. pecorum* and selected Gram-negative bacteria, the CFT does not have complete specificity [21].

Our estimates of seroprevalence in sheep flocks were at 23%, which is in line with other studies. Fayez et al. (2021) [22] estimated average seroprevalence in sheep and goat flocks at 9.6% (within the range from 1.8 to 22.9) and 9.3% (from 1.8 to 19.5), respectively, which suggests the spread of *C. abortus* infection in these animal species in Eastern Saudi Arabia [22]. This result was similar to the seroprevalence range within a flock from 3.7% to 25.0%, registered in sheep and goat in Costa Rica [23], and from 0.0% to 29.9%, registered in goats in China [24]. Such differences in seroprevalence of *C. abortus* are likely to have been caused by differences in animal breeds, farm management methods, sanitary conditions, sampling times, and serological tests in use. Moreover, antigen cross-reactivity between *C. abortus* and *C. pecorum* in sheep may result in seroprevalence overstatement [25,26,27].

Detection of Chlamydia infections improved after the introduction of immunofluorescence assays and PCR methods that enabled the direct identification on the basis of clinical samples and differentiation among *Chlamydia* species [27].

Chlamydia were detected in pathological material using the immunofluorescence assay in 60.9% of cases, and using the PCR method in 58.5% of cases.

The results described above allowed us to assume the presence of *C. abortus* infection in sheep in the Mari El Republic; however, pathogen isolation from aborted specimens (fetuses and placentas) on developing chicken embryos is the gold standard for the final diagnosis. The only downside of chlamydia isolation on chicken embryos is the need for a lengthy incubation and labor intensity [28]. In this study, 5 *Chlamydia* isolates out of 41 tested samples were selected, as they propagated well and tested positive to chlamydia inclusions in the cell culture and DNA via PCR. Apart from chicken embryos, we used a McCoy cell line, which allowed us to execute blind passaging. We attempted to decrease the probability of missing positive samples by inoculating each sample in the cell culture and completing at least three blind passages for each negative sample. So, even though chlamydia isolation on a cell culture is recognized as the most convenient method and is key to documenting organism viability [20], due to the labor intensity of chlamydia isolation from cell cultures, we cannot recommend it as a routine diagnostics’ method.

This study was further extended to identify and characterize genetic diversity of chlamydia infection in sheep in the Mari El Republic. Unfortunately, the GenBank database currently contains no sequences of Russian isolates.

Our goal was to study the *C. abortus* isolates obtained from sheep with reproduction issues, using sequencing methods for studying the *OmpA* gene (outer membrane protein of *C. abortus*). In order to resolve these issues, we present the initial analysis of a selection of sequenced *Chlamydia* species genomes that includes five *C. abortus* isolates obtained in the Mari El Republic.

We did an analysis of the dendrogram of the *OmpA* gene sequence (1058 bp). This phylogenetic analysis of the five isolates shows limited diversity of the *OmpA* gene. All DNA samples of the isolates are grouped with *C. abortus* sequences. The samples related to this study have GenBank registration numbers.

In a number of studies [29,30,31], seven sequences of the entire *C. abortus* genome were presented. *Chlamydia abortus* is a highly monomorphic group that has no conservative virulence-associated plasmid. In a 2005 study, Thomson and colleagues noted that even though clinical manifestations of chlamydial infections are diverse, whole genome sequencing showed that their genomes are remarkably conservative, and only a minor share of genomes is species-specific [30]. An analysis of available data makes it evident that the overwhelming majority of *C. abortus* isolates circulating on the international level show a low level of variation of nucleotide sequences in their genes. No recombination among sequenced isolates was identified, even though a low level of variation may impede the identification of recombination. Looking at the phylogenetic tree as a whole, we can see that all the sequenced isolates can be attributed to long branches. This type of phylogenetic structure is a common feature of phylogenetic analysis of this *Chlamydia* species that suggests either unaccounted diversity or, which is more probable, evolutionary bottle-necks. 

In a recent study, a whole genome sequencing on the collection of 57 *C. abortus* isolates was performed. The isolates originated primarily from the United Kingdom, Germany, France, and Greece, but also from Tunisia, Namibia, and the U.S. and the whole genome sequence of the British *C. abortus* strain (strain S26/3) was made available [32]. This study showed a high level of conservation of both the sequence and the entire gene content as compared to other *Chlamydiaceae*. Phylogenetic analysis of a total of 64 genomes shows a deep structural division within the *C. abortus* species with a major clade demonstrating limited diversity, in addition to a branch carrying two more distantly related Greek isolates, LLG and POS. Within the major clade, the study identified seven further phylogenetic groups, demonstrating geographical associations. The number of variable nucleotide positions across the sampled isolates is significantly lower than those published for *C. trachomatis* and *C. psittaci*. Furthermore, the *OmpA* gene (CAB048), often used for genotyping in *Chlamydia* species, also shows a very limited variation among these *C. abortus* isolates. Only seven variable nucleotide sites were found to distinguish *OmpA* in the British S26/3 strain from that in Greek LLG, and only six allelic variants of *OmpA* were evident within the whole tree [32].

Our study confirms that *C. abortus*, an infectious agent that has a major economic effect, has limited genomic variation, and that the *C. abortus* genome in the Mari El Republic of Russia appears stable. Variation in *C. abortus* isolates originating from the Mari El Republic in Russia appears much lower than that of other *Chlamydia* species within the genus. While a high identity rate detected among local strains in the framework of this study may be explained by the same host and limited territory of the study, the *C. abortus* population in Russia may be extremely stable too, with all the isolates circulating in sampled flocks belonging to the same cluster with very limited variation among isolates. This might reflect limited access to Russia, and the Mari El Republic in particular, where replacement animals are bought from local markets. This better understanding of viral diversity in Russia will potentially allow for improvement in diagnostic kits.

## 5. Benefits and Limitations

In conclusion, conventional methods of chlamydia infection diagnostics have a number of disadvantages, including low sensitivity, time consuming, and of high cost. Ratios of positive and negative samples in immunofluorescence test and in PCR test were almost even (60.9% and 58.5%, respectively). This study confirms the advantages of PCR as a fast and reliable method for highly sensitive detection and differentiation of chlamydiosis, able to simplify the *Chlamydia abortus* diagnostic in ewes. The test reliability was confirmed by DNA sequencing: all amplification products belonged to *Chlamydia abortus*. This result can be used to implement efficient measures to combat *C. abortus* infections.

Furthermore, we are continuing our research into sequencing, in particular, we are engaged in whole genome sequencing of the Chlamydia VNITIBP-21 strain. Several authors have mentioned the need for full sequencing of *chlamydia* strains as early as 2015, as they keep finding genetically mixed chlamydia, which demonstrates the importance of whole, not partial, genome analysis. At the same time, there is a study arguing that whole genome sequencing and comparative analysis showed there is no genetic basis for any attenuation of the vaccine strain [33]. We also wanted to make sure that attenuation is impossible, using whole genome sequencing of the Russian strain as an example.

Research into current strains is necessary for developing up-to-date safe and efficient vaccines. Even though there are different approaches to the development of vaccines against *C. abortus*, safe and efficient vaccines are still in deficiency [34].

## Figures and Tables

**Table 1:** Serological testing of animals for chlamydial infection.

Animal Group	Number of Animals	Number of Positive Samples (Two-Fold)
Stud rams	10	1
Lambers	10	4
Lambs (2–3 months old)	10	2
Total, heads (%)	30	7 (23%)

**Table 2:** Analysis of pathological material of aborted fetuses and placentas for chlamydial infection.

Sample Source	Total Samples	Immunofluorescence Assay	Isolation on Chicken Embryos	Real-Time PCR **
Fetal lungs	12	6	6 (1 *)	6
Ovine placentas	12	8	8 (1 *)	7
Fetal liver	5	2	2 (1 *)	2
Fetal spleen	7	4	4 (1 *)	4
Vaginal swabs	5	5	5 (1 *)	5
Total samples (%)	41	25 (60.9%)	25 (60.9%)	24 (58.5%)

* isolates were selected due to their high reproduction capability and were positive in all used assays; ** numbers given represent probes confirmed positive by both in-house and commercial PCR tests.

**Table 3:** Description of collected *C. abortus* isolates.

Isolate Name	Isolation Area	Sample	Number of Passages on Chicken Embryo	Number of Passages on Cell Culture
Chlamydia Mari El 21-1	Volzhsk district	Fetal lungs	3	16
Chlamydia Mari El 21-2	Sernur district	Vaginal swabs	3	14
Chlamydia Mari El 21-3	Sovetsky district	Fetal liver	8	13
Chlamydia Mari El 21-4	Orshanka district	Placenta	4	13
Chlamydia VNITIBP-21	Medvedevo district	Fetal spleen	5	20

**Table 4:** Homology of the Chlamydia VNITIBP-21 strain to strains closely related via the *OmpA* gene. The isolates selected and sequenced in this study are written in red.

Strain Name	GenBank Accession Code	Year of Isolation	Country	Homology Value
GT	JF728987.1	2011	Greece	99.8
LADEAP-A-253	KX011470.1	2016	Argentina	98.8
** **Chlamydia Mari El 21-3** **	OP414762	2021	Russia	99.8
C18/98	DQ435300.1	2006	The Netherlands	99.7
S26/3	CR848038.1	2006	United Kingdom	99.7
GIMC 2006	NZ_CP024084.1	2017	USA	99.7
** **Chlamydia Mari El 21-1** **	OP414760	2021	Russia	99.7
TB	JF728988.1	2011	Greece	99.7
0801	GU320570.1	2009	China	99.7
PS	JF728984.1	2011	Greece	99.7
** **Chlamydia Mari El 21-2** **	OP414761	2021	Russia	99.5
CA0302	AB239904.1	2005	Japan	99.5
CH1	LC557139.1	2020	Egypt	99.5
SH1	LC554223.1	2020	Egypt	99.5
CEDIVE-G-4661/12	KU728158.1	2016	Argentina	99.4
** **Chlamydia Mari El 21-4** **	OP414763	2021	Russia	99.3

## Data Availability

All data generated or analyzed during this study are included in the manuscript and Supplementary Materials. All of the study’s original images are included in the manuscript. The sequences of the OmpA gene are available in the GenBank repository (https://www.ncbi.nlm.nih.gov/genbank/ (accessed on 28 Jun 2022) for Chlamydia VNITIBP-21 isolate and on 13 Sep 2022 for Chlamydia Mari El 21-1, Chlamydia Mari El 21-2, Chlamydia Mari El 21-3 and Chlamydia Mari El 21-4 isolates).

## Supplementary Materials

The following supporting information can be downloaded at: https://www.mdpi.com/article/10.3390/pathogens11121408/s1, Figure S1: Location of the five sheep flocks selected for the study on the map of the Mari El Republic (marked and numbered with red): 1—Chlamydia Mari El 21-1 (Volzhsk district), 2—Chlamydia Mari El 21-2 (Sernur district); 3—Chlamydia Mari El 21-3 (Sovetsky district), 4—Chlamydia Mari El 21-4 (Orshanka district); 5—Chlamydia VNITIBP-21 (Medvedevo district); Figure S2: Comparison of nucleotide sequences of Chlamydia VNTIBP-21 of 20th (McCoy cell culture) and 2nd (yolk sac) passages.
