# Supplementary material for: Chlamydia abortus Isolation and Identification in Aborted Ovine Fetus in Mari El Republic of Russia
Source: Pathogens. 2022 Nov 24;11(12):1408. doi: 10.3390/pathogens11121408 (PMC9784139; doi:10.3390/pathogens11121408)
Supplement: Supplementary file 1 [file pathogens-11-01408-s001.zip › pathogens-1978687-supplementary.pdf]

Supplementary Information

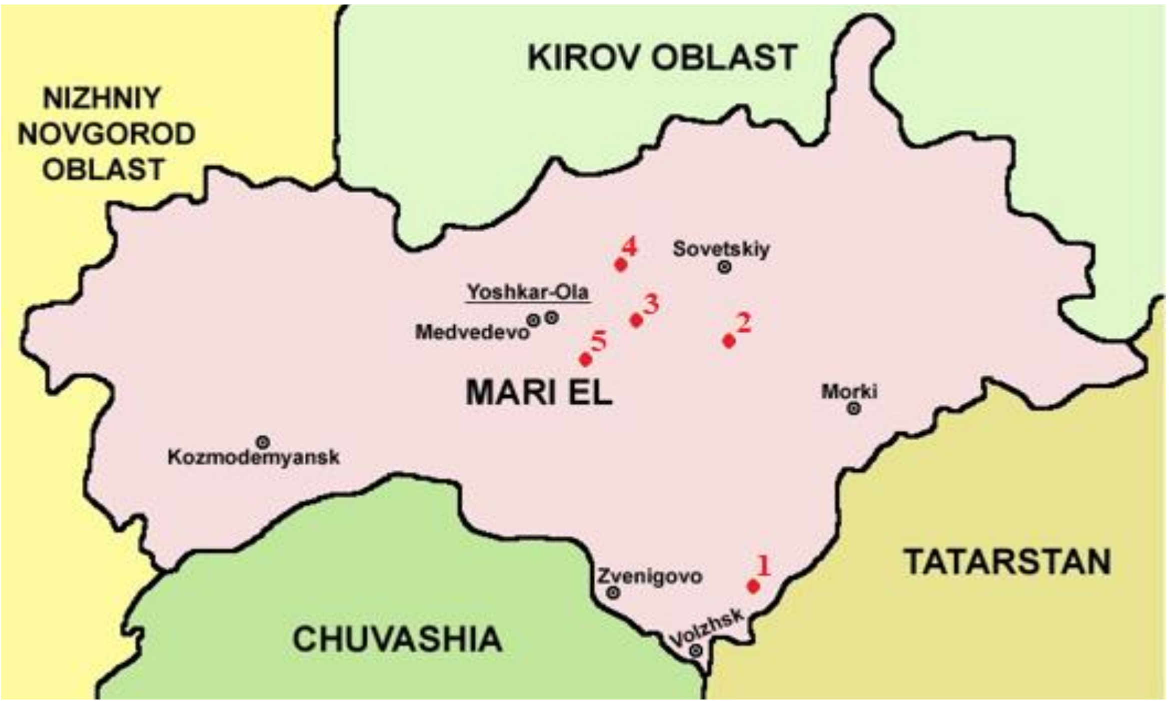

Figure S1. Location of the five sheep flocks selected for the study on the map of the Mari El Republic (marked and numbered with red): **1** - Chlamydia Mari El 21-1 (Volzhsk district), **2** - Chlamydia Mari El 21-2 (Sernur district); **3** - Chlamydia Mari El 21-3 (Sovetsky district), **4** - Chlamydia Mari El 21-4 (Orshanka district); **5** - Chlamydia VNITIBP-21 (Medvedevo district).

```
Score              Expect   Identities       Gaps          Strand
1613 bits(873)     0.0      873/873(100%)    0/873(0%)     Plus/Plus

Query  1    ATCGATGGCACTATGTGGGAAGGTGCTTCAGGTGATCCTTGCGATCCTTGCTCTACTTGG  60
            ||||||||||||||||||||||||||||||||||||||||||||||||||||||||||||
Sbjct  1    ATCGATGGCACTATGTGGGAAGGTGCTTCAGGTGATCCTTGCGATCCTTGCTCTACTTGG  60

Query  61   TGTGATGCTATCAGCATCCGCGCAGGATACTACGGAGATTATGTTTTCGATCGTGTATTA  120
            ||||||||||||||||||||||||||||||||||||||||||||||||||||||||||||
Sbjct  61   TGTGATGCTATCAGCATCCGCGCAGGATACTACGGAGATTATGTTTTCGATCGTGTATTA  120

Query  121  AAAGTTGATGTGAATAAAACTATCACCGGCATGGGTGCAGTTCCTACAGGAACCGCAGCA  180
            ||||||||||||||||||||||||||||||||||||||||||||||||||||||||||||
Sbjct  121  AAAGTTGATGTGAATAAAACTATCACCGGCATGGGTGCAGTTCCTACAGGAACCGCAGCA  180

Query  181  GCTAATTACAAAACTCCTACGGATAGACCCAACATCGCTTACGGCAAACACTTACAAGAC  240
            ||||||||||||||||||||||||||||||||||||||||||||||||||||||||||||
Sbjct  181  GCTAATTACAAAACTCCTACGGATAGACCCAACATCGCTTACGGCAAACACTTACAAGAC  240

Query  241  GCCGAATGGTTCACCAATGCAGCTTTCCTCGCATTGAATATCTGGGATCGCTTTGATATT  300
            ||||||||||||||||||||||||||||||||||||||||||||||||||||||||||||
Sbjct  241  GCCGAATGGTTCACCAATGCAGCTTTCCTCGCATTGAATATCTGGGATCGCTTTGATATT  300

Query  301  TTCTGCACATTAGGCGCTTCTAATGGGTACTTCAAAGCTAGTTCTGCGCCATTCAACCTC  360
            ||||||||||||||||||||||||||||||||||||||||||||||||||||||||||||
Sbjct  301  TTCTGCACATTAGGCGCTTCTAATGGGTACTTCAAAGCTAGTTCTGCGCCATTCAACCTC  360

Query  361  GTTGGTTTGATTGGTGTTAAAGGATCCTCCATAGCAGCTGATCAGCTTCCCAATGTAGGC  420
            ||||||||||||||||||||||||||||||||||||||||||||||||||||||||||||
Sbjct  361  GTTGGTTTGATTGGTGTTAAAGGATCCTCCATAGCAGCTGATCAGCTTCCCAATGTAGGC  420

Query  421  ATCACTCAAGGAATCGTTGAATTTTATACAGATACAACATTCTCTTGGAGTGTAGGTGCA  480
            ||||||||||||||||||||||||||||||||||||||||||||||||||||||||||||
Sbjct  421  ATCACTCAAGGAATCGTTGAATTTTATACAGATACAACATTCTCTTGGAGTGTAGGTGCA  480

Query  481  CGCGGAGCTTTATGGGAGTGTGGTTGTGCGACTTTAGGAGCAGAGTTCCAATACGCTCAG  540
            ||||||||||||||||||||||||||||||||||||||||||||||||||||||||||||
Sbjct  481  CGCGGAGCTTTATGGGAGTGTGGTTGTGCGACTTTAGGAGCAGAGTTCCAATACGCTCAG  540

Query  541  TCTAATCCTAAAATTGAAATGTTGAATGTAGTCTCCAGCCCAGCACAATTTGTGGTTCAC  600
            ||||||||||||||||||||||||||||||||||||||||||||||||||||||||||||
Sbjct  541  TCTAATCCTAAAATTGAAATGTTGAATGTAGTCTCCAGCCCAGCACAATTTGTGGTTCAC  600

Query  601  AAGCCTAGAGGATACAAGGGAACAGCATTTCCTTTACCTCTAACAGCTGGTACTGATCAG  660
            ||||||||||||||||||||||||||||||||||||||||||||||||||||||||||||
Sbjct  601  AAGCCTAGAGGATACAAGGGAACAGCATTTCCTTTACCTCTAACAGCTGGTACTGATCAG  660

Query  661  GCAACTGACACTAAGTCGGCTACAATTAAATACCACGAATGGCAAGTTGGTTTAGCGCTC  720
            ||||||||||||||||||||||||||||||||||||||||||||||||||||||||||||
Sbjct  661  GCAACTGACACTAAGTCGGCTACAATTAAATACCACGAATGGCAAGTTGGTTTAGCGCTC  720

Query  721  TCGTATCGATTGAACATGCTTGTTCCTTACATTGGCGTAAACTGGTCACGAGCAACTTTT  780
            ||||||||||||||||||||||||||||||||||||||||||||||||||||||||||||
Sbjct  721  TCGTATCGATTGAACATGCTTGTTCCTTACATTGGCGTAAACTGGTCACGAGCAACTTTT  780

Query  781  GATGCTGACGCTATCCGCATCGCTCAACCTAAATTAGCTGCTGCTGTGTTAAACTTGACC  840
            ||||||||||||||||||||||||||||||||||||||||||||||||||||||||||||
Sbjct  781  GATGCTGACGCTATCCGCATCGCTCAACCTAAATTAGCTGCTGCTGTGTTAAACTTGACC  840

Query  841  ACATGGAACCCAACCCTTTTAGGAGAAGCTACA  873
            |||||||||||||||||||||||||||||||||
Sbjct  841  ACATGGAACCCAACCCTTTTAGGAGAAGCTACA  873
```

Figure S2. Comparison of nucleotide sequences of Chlamydia VNTIBP-21 of 20th (McCoy cell culture) and 2nd (yolk sac) passages
